# The impact of COVID-19 on patients with epilepsy

**DOI:** 10.1590/0004-282X-ANP-2020-0517

**Published:** 2021-05-07

**Authors:** Gloria Maria de Almeida Souza TEDRUS, João Fernando Cloclet Pio da SILVA, Gabriel Santaterra BARROS

**Affiliations:** 1 Pontifícia Universidade Católica de Campinas, Programa de Pós-Graduação em Ciências da Saúde, Campinas SP, Brazil. Pontifícia Universidade Católica de Campinas Pontifícia Universidade Católica de Campinas Programa de Pós-Graduação em Ciências da Saúde Campinas SP Brazil; 2 Pontifícia Universidade Católica de Campinas, Faculdade de Medicina, Campinas SP, Brazil. Pontifícia Universidade Católica de Campinas Pontifícia Universidade Católica de Campinas Faculdade de Medicina Campinas SP Brazil

**Keywords:** Coronavirus Infections, Pandemics, Epilepsy, Seizures, Infecções por Coronavirus, Pandemias, Epilepsia, Convulsões

## Abstract

**Background:**

The COVID-19 pandemic and social distancing can have adverse impacts on adult people with epilepsy (PWE).

**Objective:**

To investigate the seizure frequency, the perceived well-being, and the presence of anxiety symptoms in PWE during the COVID-19 pandemic period.

**Methods:**

Data from a questionnaire on the repercussions of COVID-19 were analyzed in relation to the clinical variables of 114 PWE, with a significance level of p<0.05.

**Results:**

There were 26 cases of COVID-19 in PWE and/or family members (22.8%). During the pandemic period, 11 PWE (9.6%) reported an increase in seizures, but unrelated to COVID-19. Also, the number of crises in PWE with previous depressive disorders increased, with differences between epilepsies. Symptoms of depression, impaired well-being, and concern for their lifestyle were significant in PWE with a previous diagnosis of depression. Impaired well-being, increased anxiety, nervousness, and tiredness, and the concern with being infected were mentioned by a high number of PWE in the pandemic.

**Conclusion:**

Seizure frequency increased during the pandemic period, a finding associated with clinical variables of epilepsy. PWE with depression had worse perceived well-being. Changes in well-being and increased anxiety and nervousness were frequent in the pandemic.

## RESUMO

**Introdução:**

A pandemia por COVID-19 e o isolamento social podem ter impactos adversos em pessoas adultas com epilepsia (PCE).

**Objetivo:**

Investigar a frequência das crises, a percepção de bem-estar e a presença de sintomas de ansiedade em PCE no período da pandemia da COVID-19.

**Métodos:**

Foram relacionados os dados de um questionário de repercussões da COVID-19 com as variáveis clínicas de 114 PCE, com nível de significância p<0,05.

**Resultados:**

A COVID-19 ocorreu em 26 (22,8%) PCE e/ou familiares. Durante o período da pandemia, houve aumento das crises epilépticas em 11 (9,6%) PCE, mas sem relação com o diagnóstico de COVID-19. No período da pandemia, houve aumento do número de crises nos PCE com transtorno depressivo prévio e de modo distinto entre as epilepsias. Sintomas de depressão, comprometimento do bem-estar e preocupação com o estilo de vida ocorreram significativamente nos PCE com diagnostico anterior de depressão. O comprometimento do bem-estar, o aumento da ansiedade, do nervosismo e do cansaço, e a preocupação em contrair a doença foram referidos na maior parte do tempo em elevado número de PCE na pandemia.

**Conclusão:**

Houve aumento da ocorrência de crises epilépticas durante o período pandêmico e isso foi associado a variáveis clínicas da epilepsia. Pior percepção de bem-estar ocorreu nos PCE com depressão. Alteração do bem-estar e aumento de ansiedade e de nervosismo foram frequentes na pandemia.

## INTRODUCTION

The coronavirus disease 2019 (COVID-19) is caused by the novel coronavirus (SARS-CoV-2), which was initially identified in China but quickly became a pandemic in early 2020, with great potential for transmission^1^. The lack of vaccines and specific therapeutic alternatives led to the encouragement of social distancing and better self-care as a way to control the spread of the disease, with consequent ethical dilemmas, as well as economic and social impacts^1^^,^^2^. In pandemics, the fear of getting the disease and the measures to restrict social contact can have a major effect on each person’s lifestyle, with negative psychological repercussions for the population^3^.

The pandemic has accentuated the vulnerability of individuals in unfavorable social conditions, particularly in countries with poor socioeconomic and cultural development. In Brazil, the Unified Health System (*Sistema Único de Saúde* - SUS), a public and free health care system, sought to offer greater medical support to the growing number of patients with COVID-19 and reorganized the admission of patients to the health care system, in detriment to providing support to other acute and chronic conditions^4^.

Epilepsy is a chronic neurological disease with a high incidence and prevalence, associated with psychiatric comorbidities in a high number of cases. To date, no evidence has been found of associations between epilepsy and COVID-19^5^. However, this study is justified because it seeks to investigate demographic and clinical aspects related to the pandemic context and its association with symptoms of anxiety and changes in the lifestyle of adult people with epilepsy (PWE).

Thus, this study aimed to verify the seizure frequency, the perceived well-being, and the occurrence of anxiety symptoms in PWE residing in the city of Campinas, São Paulo, Brazil, during the COVID-19 pandemic period.

## METHODS

### Patients

A total of 114 consecutive PWE older than 18 years were invited to participate in this study in their routine medical visit to the outpatient clinic of clinical neurology at the Pontifícia Universidade Católica de Campinas (PUC-Campinas), Campinas, Brazil, during the pandemic and social distancing period, between August 1 and September 10, 2020. Prevention and protection protocols against SARS-CoV-2 were instituted for professionals, PWE, and family members. Epilepsy was diagnosed according to the International League Against Epilepsy (ILAE) Classification of the Epilepsies^6^. Patients who had difficulty understanding the questions in the instrument due to a lower educational level or mental disabilities were excluded. The study was approved by the Human Research Ethics Committee of PUC-Campinas.

Campinas is a city in Southeastern Brazil with a population of 1,080,113 inhabitants. In Campinas, the first case of COVID-19 was reported on March 13, with a total of 30,674 confirmed cases by September 15, 2020.

### Procedures

A questionnaire was administered to collect sociodemographic (age, sex, educational level) and clinical data (age at onset, seizure type and frequency, number of antiepileptic drugs taken). The psychiatric service assessed the PWE with depressive symptoms, diagnosed with recurrent depressive disorder (according to the international classification of diseases) prior to the pandemic period.

The participants were questioned whether they or their family members who live in the same residence had been diagnosed with COVID-19 (clinically and tested for SARS-CoV-2, by reverse transcription-polymerase chain reaction [RT-PCR]).

They were asked about the repercussions felt during the pandemic, with the administration of a specific ILAE questionnaire, available at the ILAE website^7^. These questions included:

- aspects of epilepsy (changes in seizure frequency, difficulty in obtaining AEDs, and access to health services);
- Likert questions (always - never) about their perceived well-being (nervous, hopeless, tired or restless, depressed - nothing can cheer me up, everything is an effort, useless);
- Likert questions (yes - no) about aspects of self-reported anxiety (concern with being infected, with the quarantine, with interferences in family and social life, with a financial aspect);
- a question about whether the person had any specific support needs.

Descriptive data analysis was performed. Continuous variables were expressed as mean and standard deviation (SD). Categorical variables were reported as numbers and percentages. Data were processed by the IBM SPSS Statistics software, version 22. The significance level was set at 5% (p<0.05).

## RESULTS

Table 1 shows demographic data and epilepsy aspects of 114 PWE, as well as their seizure frequency and special needs during the pandemic period.

**Table 1.** Demographic and clinical data on epilepsy, seizure frequency, and the perceived needs of 114 PWE in the pandemic period.

	n, %, or mean±SD
Age (years)	45.8 (±16.2)
Educational level (years)	5.9 (±4.0)
Sex-female	56 (49.1%)
Age at epilepsy onset (years)	23 (±18.8)
Seizure frequency	
≥1 seizure/month	39 (34.2%)
1-11 seizures/last year	31 (27.2%)
No seizures in the last year	44 (38.6%)
Number of AEDs taken: one	70 (61.4%)
Depression in the period before the pandemic	30 (26.3%)
Epilepsy syndrome	
Genetic	6 (5.3%)
Structural	79 (69.3%)
Unknown etiology	29 (25.4%)
Subjective perception in the pandemic	
Seizure frequency	
Unaltered	99 (86.8%)
Increase	11 (9.6%)
Decrease	4 (3.5%)
Difficulty in obtaining AEDs	47 (41.2%)
Difficulty in contacting health professionals	34 (29.8%)
Special needs in the period	
Medical support and access to AEDs	56 (49.1%)
Psychological support	25 (21.9%)
Economic, financial, and dietary problems	20 (17.5%)
Trusted information about COVID-19	15 (13.2%)

COVID-19: coronavirus disease 2019; PWE: people with epilepsy; AED: antiepileptic drugs, SD: standard deviation.

COVID-19 was diagnosed by the clinical manifestation associated with being RT-PCR-positive for COVID-19 in three PWE and 13 family members, and only by mild/moderate clinical symptoms in nine PWE and seven family members.

Nervousness, depressive symptoms, tiredness or restlessness, and concern about being infected with SARS-CoV-2 were reported by a high number of PWE (always or most of the time during the pandemic). Complaints about changes in well-being and increased anxiety occurred less often (Table 2).

**Table 2.** Perceived well-being and aspects of self-reported anxiety by PWE during the COVID-19 pandemic period.

How are you feeling?	Always or part of the time	Never
Nervous	70 (61.4%)	44 (38.6%)
Hopeless	34 (29.8%)	80 (70.2%)
Tired or restless	61 (53.5%)	53 (46.5%)
Depressed - nothing can cheer me up	41 (36%)	73 (64%)
Everything is an effort	38 (33.3%)	76 (66.7%)
Useless	25 (21.9%)	89 (78.1%)
Anxiety aspects	Yes	No
Are you worried about getting the disease?	59 (51.8%)	55 (48.2%)
Are you worried about the quarantine?	37 (32.5%)	77 (67.5%)
Are you worried about interrupting your family or social life due to the lockdown?	47 (41.2%)	67 (58.8%)
Are you worried about a higher seizure frequency or severity?	46 (40.4%)	68 (59.6%)
Are you worried about financial matters?	42 (36.8%)	72 (63.2%)
Are you worried about lifestyle changes?	38 (33.3%)	76 (66.7%)
Are you worried about having to take care of other people?	33 (28.9%)	81 (71.1%)

COVID-19: coronavirus disease 2019; PWE: people with epilepsy.

During the pandemic period, concerns about the increase in the number or severity of seizures, the perceived well-being, and the presence of self-reported anxiety symptoms were significantly associated with several symptoms of epilepsy and the occurrence of COVID-19 in family members (Table 3).

**Table 3.** Perceived well-being and anxiety aspects of PWE during the pandemic period according to the incidence of COVID-19 and the clinical variables of epilepsy.

	Worried about a higher seizure frequency or severity		
	Yes (n=46)	No (n=68)	p-value
Family member with COVID-19			
No	42	52	0.041*
Yes	4	16	
PWE with COVID-19			
No	8	60	0.600
Yes	42	4	
Seizure frequency before the pandemic			
≥1 seizure/month (n=39)	21	18	0.034*
<1 seizure/month (n=75)	25	50	
	**Depressed - nothing can cheer me up**		
	**Yes (n=41)**	**No (n=73)**	**p-value**
Number of AEDs taken			
One	20	50	0.038*
≥2	21	23	
Depression in the period before the pandemic			
No	13	60	0.006*
Yes	17	13	
	**Worried about being useless**		
	**Yes (n=25)**	**No (n=89)**	**p-value**
Depression in the period before the pandemic			
No	12	72	0.001*
Yes	13	17	
	**Worried about lifestyle changes**		
	**Yes (n=38)**	**No (n=76)**	**p-value**
Seizure frequency before the pandemic			
≥1 seizure/month (n=39)	18	21	0.036*
<1 seizure/month (n=75)	20	55	

COVID-19: coronavirus disease 2019; PWE: people with epilepsy; AED: antiepileptic drugs; * p<0.05.

The increased number of seizures in the pandemic was associated with the type of epilepsy, a previous diagnosis of depressive disorder, and the perception of nervousness during this period (Table 4).

**Table 4.** Clinical variables according to seizure frequency in the COVID-19 pandemic.

	Increase in the number of seizures in the COVID-19 pandemic		
	No (n=103)	Yes (n=11)	p-value
PWE with COVID-19			
No	93	9	0.326^a^
Yes	10	2	
Epilepsy			
Genetic	3	3	0.001^b^*
Unknown etiology	25	4	
Structural	75	4	
Seizure frequency before the pandemic			
≥1 seizure/month	33	6	0.182^a^
<1 seizure/month	70	5	
Depression in the period before the pandemic			
No	79	5	0.036^a^*
Yes	24	6	
Perceived nervousness in the pandemic period			
No	35	9	0.003^a^*
Yes	68	2	
Worried about a higher seizure frequency or severity			
No	62	6	0.754^b^
Yes	41	5	

COVID-19: coronavirus disease 2019; PWE: people with epilepsy; ^a^: Fisher’s exact test; ^b^: chi-square test; *p<0.05.

## DISCUSSION

This study sought to investigate the clinical variables and the perceived well-being, aspects of anxiety, and self-reported special needs related to the context of the COVID-19 pandemic by PWE who were regularly monitored in a clinical neurology clinic in the city of Campinas, Brazil.

In this study, the seizure frequency increased by 9.6% of PWE, unrelated to a confirmed diagnosis of COVID-19 in PWE and/or family members. Similar values were reported in a study with 189 Italian patients, conducted through telephone interviews^8^. We found a higher seizure frequency in PWE with genetic epilepsy, a previous diagnosis of depression, and who self-reported nervousness during the pandemic period, which may suggest the emotional factor as a seizure trigger. Studies have described an association of greater seizure frequency - in approximately 30% of cases - with high levels of stress^9^^,^^10^ and seizure severity or poor adherence to treatment^9^. However, the effect of the pandemic on the occurrence of seizures remains unclear^8^^,^^11^.

In the pandemic, PWE with a previous diagnosis of depressive disorder experienced symptoms of depression, overexertion, and uselessness, as well as increased financial worries and changes in lifestyle, suggesting that psychological distress in this period can worsen a previous mental health condition. This finding led us to infer that PWE are more vulnerable to stress associated with a pandemic, as already described in studies of other chronic diseases^12^.

Complaints of nervousness, negative emotions, and concerns about getting COVID-19 were frequent in PWE. Hao et al. reported that PWE with refractory epilepsy experience more psychosocial distress compared to healthy individuals^13^. However, other authors found that the anxiety level of PWE was not significantly higher than that of the general population^14^.

The concern with medical support, the worry of seeking medical care due to the fear of exposure to SARS-CoV-2, the cancellation of regular medical treatments, the need to have psychological support and reliable information about COVID-19, and the difficulty in obtaining AEDs due to restrictions in their distribution can contribute to the deterioration in PWE psychological and physical stress during the pandemic. In Brazil, the mental health impairment of PWE can be aggravated by social inequality, deficiencies in health services, and the poor socioeconomic and cultural conditions of a high proportion of the population.

The present study has limitations. First, our study is cross-sectional, which prevents us from investigating causal relationships. Our study took place in a single medical center in a country with specific cultural and population characteristics, making it difficult to generalize these findings. Aspects of well-being and the presence of anxiety were self-reported, which can lead to the risk of bias. Our data must be compared to samples from other research centers.

In conclusion, a higher seizure frequency was identified during the pandemic, associated with clinical variables of epilepsy. PWE with depression had worse perceived well-being. Changes in well-being, increased anxiety, as well as the need for medical, psychological, and financial support, for access to AEDs, and for reliable information about COVID-19, were frequent in PWE.
